# Comparative Effectiveness of Antidiabetic Drugs as an Additional Therapy to Metformin in Women with Polycystic Ovary Syndrome: A Systematic Review of Metabolic Approaches

**DOI:** 10.1155/2024/9900213

**Published:** 2024-03-11

**Authors:** Maryam Heidarpour, Mehrzad Mojarad, Sadegh Mazaheri-Tehrani, Ali Kachuei, Arash Najimi, Davood Shafie, Hassan Rezvanian

**Affiliations:** ^1^Isfahan Endocrine and Metabolism Research Center, Isfahan University of Medical Sciences, Isfahan, Iran; ^2^Child Growth and Development Research Center, Research Institute for Primordial Prevention of Non-Communicable Disease, Isfahan University of Medical Sciences, Isfahan, Iran; ^3^Heart Failure Research Center, Cardiovascular Research Institute, Isfahan University of Medical Sciences, Isfahan, Iran; ^4^Medical Education Department, Medical Education Research Center, Education Development Center, Isfahan University of Medical Sciences, Isfahan, Iran

## Abstract

**Background:**

Metformin is commonly prescribed to treat polycystic ovary syndrome (PCOS) patients, but in some cases, it may not be effective even at high doses or may cause intolerable side effects. Therefore, recent studies have examined the impact of combining metformin with other antidiabetic medications.

**Methods:**

A systematic search was performed in Scopus, PubMed, Web of Science, and Embase up to 30 June 2023. All interventional studies that assessed the efficacy of different antidiabetic agents were included.

**Results:**

Among the 3488 records found in the primary search, 16 papers were included. Our study showed that dipeptidyl peptidase-4 inhibitors (DPP4i) had the most significant impact on glycemic profile, while thiazolidinediones (TZDs) had the most influence on lipid levels. However, it was observed that patients taking only metformin experienced a greater increase in high-density lipoprotein cholesterol (HDL-C) levels. Glucagon-like peptide-1 receptor agonists (GLP1RAs) effectively modified various anthropometric measurements, such as weight, body mass index, waist circumference, and waist-to-hip ratio. The effects of different antidiabetic drugs on hormone levels were inconclusive, although testosterone levels were more affected by GLP1RA, sodium-glucose cotransporter-2 inhibitors (SGLT2i), and TZDs. None of the combined therapies showed a significant change in blood pressure.

**Conclusion:**

Since PCOS is a metabolic disorder, choosing the best combination of antidiabetic drugs in the clinical course of PCOS patients will be very important. Today, it seems that we need a new metabolic approach for better treatment of the metabolic aspects of these patients.

## 1. Introduction

Polycystic ovary syndrome (PCOS) is a common disorder among females of reproductive age, with an estimated prevalence of 4–20% worldwide [1, 2]. It is characterized by different metabolic and hormonal abnormalities such as oligoovulation or anovulation, hyperandrogenism, insulin resistance (IR), type 2 diabetes mellitus (T2DM), obesity, dyslipidemia, and ultrasound findings including polycystic ovary [3, 4]. Rotterdam criteria are the commonly known statements for diagnosis of PCOS. It is defined if any two items of the following are present: first evidence of oligoovulation or anovulation, second biochemical or clinical hyperandrogenism, and third polycystic ovarian morphology on ultrasound, with the exclusion of other relevant disorders [5]. IR is one of the common metabolic disorders among PCOS patients, with a frequency of approximately 35–80%, independent of the body fat distribution or being obese. IR makes PCOS patients more likely to develop further complications such as T2DM [6, 7]. According to the Centers for Disease Control and Prevention (CDC), more than half of PCOS patients develop T2DM by age 40 [8]. Since the exact pathophysiology behind PCOS has not yet been well understood, most available therapies are symptomatic, and few medications have been established for hormonal and metabolic dysregulations [4, 9].

Metformin, from the family of biguanides, is usually prescribed as the first-line drug for modifying the metabolic features of PCOS, including obesity, IR, impaired glucose metabolism, and T2DM [4, 10]. Metformin exerts its therapeutic effects by diminishing glucose production in the liver, inhibiting gluconeogenesis and lipogenesis, and increasing insulin sensitivity across peripheral tissues [11]. Besides the metabolic parameters, metformin therapy demonstrated a significant impact on lowering the total testosterone, 17-hydroxyprogesterone, androstenedione, and low-density lipoprotein cholesterol (LDL-C) and increasing the possibility of pregnancy among PCOS patients [12]. However, some cases do not respond effectively to metformin monotherapy, even at the highest dose, and others cannot tolerate its side effects. The most common side effect of metformin is gastrointestinal discomfort, such as nausea, vomiting, diarrhea, and abdominal pain [13, 14]. Thus, recent studies have assessed the effect of other antidiabetic drugs in combination with metformin [15, 16]. Here, we conducted a systematic review to find the studies that evaluated the efficacy of hypoglycemic drugs, including dipeptidyl peptidase-4 inhibitors (DPP4i), sodium-glucose cotransporter-2 inhibitors (SGLT2i), thiazolidinediones (TZDs), and glucagon-like peptide-1 receptor agonists (GLP1RAs) as a combination therapy with metformin. Moreover, we will discuss the preference of each add-on medication regarding its effect on lipid profile, anthropometric measures, sexual hormones, glucose metabolism, IR, and blood pressure.

## 2. Methods

### 2.1. Search Strategy

This study was planned, performed, and reported based on the Preferred Reporting Items for Systematic Reviews and Meta-Analyses (PRISMA) statement [17]. The protocol of this systematic review was registered at PROSPERO (CRD42023462716). We systematically searched databases, including Scopus, MEDLINE (PubMed), Web of Science, and Embase, for articles published up to the end of June 2023. The search string used keywords such as metformin, polycystic ovary syndrome, and clinical trial. The details of each database's search line are shown in Supplementary File 1.

### 2.2. Inclusion and Exclusion Criteria

All randomized clinical trials (RCTs) that assessed the effect of metformin in combination with other antidiabetic drugs on anthropometric, metabolic, and hormonal parameters among PCOS patients were included. There is no restriction for blindness, follow-up duration, race, country, and publication year. Only English articles were included. We excluded the studies that assumed other drugs as the main intervention and assessed the effect of metformin as an add-on medication. Duplicate records, conference proceedings, *in vivo* and *in vitro* experiments, and studies with insufficient data or poor quality were also excluded.

### 2.3. Screening Process and Data Extraction

Two reviewers (MM and SM-T) independently screened the primary results of the literature review according to the predetermined criteria for inclusion and exclusion. The following information was extracted from the eligible articles by two independent reviewers (MH and MM): first author, date of publication, country, the exact design of the study (blindness and arms of the trial), demographic characteristics of the participants, intervention and the duration of it, dose of the consumed medications, and the outcome of the patients. Any disagreement surrounding the screening process or data extraction was resolved by consultation with the third reviewer (HR).

### 2.4. Quality Assessment

The quality of the included articles was evaluated using the National Institute of Health (NIH) quality assessment tool for controlled intervention studies [18]. This scale consists of 14 questions and qualifies studies as poor, fair, or good. Two independent reviewers (MH and SM-T) assessed the quality of the studies, and controversies were reconciled via consensus with the third reviewer (HR).

## 3. Results

### 3.1. Study Selection

**Figure 1:**
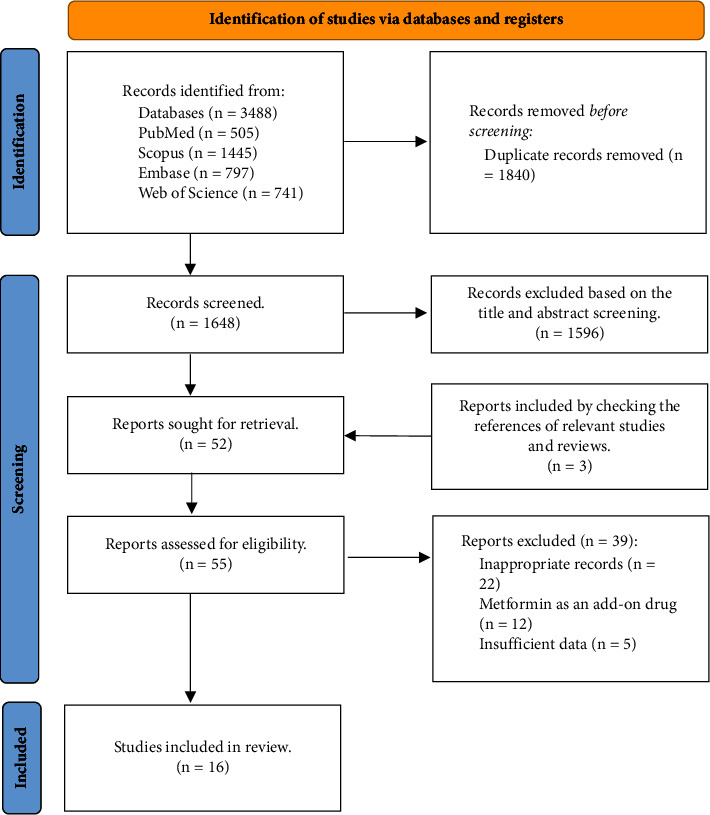
PRISMA flow diagram.

A total of 3488 records were found from the primary search in the mentioned databases. After duplicate removal, 1648 reports remained. According to the title and abstract screening, 52 articles were eligible for further assessment through the full text. Finally, 16 RCTs were eligible for inclusion in the systematic review.  Figure 1 demonstrates the study selection process.

### 3.2. Study Characteristics

The details of the 16 included studies are summarized in Table 1. A total of 878 PCOS patients were investigated between 2004 and 2023. The lowest and highest sample sizes of the included studies were 23 [23] and 137 [15], respectively. Most of the included studies have used the Rotterdam criteria for diagnosing PCOS. Included studies utilized different levels of blindness as follows: 13 reports open-label, 1 single-blind, and 2 double-blind. The duration of the intervention varied from 8 weeks to 24 weeks. In all included citations, the control group consumed different dosages of metformin varying from 850 to 2000 mg per day. On the other hand, in most cases, for the intervention group, an antidiabetic drug was added to the same dosage of metformin that had been consumed in the control group. The impact of different antidiabetic agents, including DPP4i, SGLT2i, GLP1RA, and TZDs, on glycemic and lipid profiles, anthropometric measures, and sexual hormones was investigated. All investigations in this review were single-center assays from different countries, including China [15, 26–32], Iran [16, 21], Slovenia [24, 25], the USA [22, 23], Pakistan [19], and Venezuela [20].

According to the NIH quality assessment tool, 12 out of 16 included studies in this review qualified as good and 4 as fair. The details of the quality assessment process are demonstrated in Supplementary File 2.

According to the available literature, the low number of studies on each add-on medication, on the one hand, and the great heterogeneity of the included studies due to different patients' conditions, on the other hand, persuade us not to conduct a meta-analysis.

### 3.3. Effects of Antidiabetic Drugs as an Add-On Medication to Metformin on Glycemic Profile

Fifteen reports out of 16 included studies have assessed the changes in fasting blood sugar (FBS) [15, 16, 19, 20, 23, 26–28, 30–32] or homeostatic model assessment of insulin resistance (HOMA-IR) [15, 16, 19, 21–28, 30–32]. Effects of different agents, including pioglitazone, saxagliptin, rosiglitazone, exenatide, beinaglutide, liraglutide, and canagliflozin on both FBS and HOMA-IR, have been evaluated. Besides, two studies have examined the effect of sitagliptin on HOMA-IR [24, 25]. The details of the glycemic profile alterations are shown in Table 2.

### 3.4. Effects of Antidiabetic Drugs as an Add-On Medication to Metformin on Anthropometric Measures

Fourteen included reports have investigated the alterations in different anthropometric measures, including body weight (BW) [15, 16, 19, 20, 22, 24, 25, 27–32], body mass index (BMI) [15, 16, 19, 20, 22–25, 27–32], waist circumference (WC) [15, 23–25, 27, 28, 30], and waist-to-hip ratio (WHR) [15, 16, 20, 28, 30]. The impact of different drugs, including pioglitazone, sitagliptin, rosiglitazone, exenatide, saxagliptin, beinaglutide, liraglutide, and canagliflozin as an add-on drug, has been assessed. Table 2 summarizes the details of anthropometric changes.

### 3.5. Effects of Antidiabetic Drugs as an Add-On Medication to Metformin on Lipid Profile

Twelve included records have considered the changes in different lipid profile components including triglycerides (TG) [15, 16, 22–30, 32], total cholesterol (TC) [15, 16, 22–25, 27–30, 32], high-density lipoprotein cholesterol (HDL-C) [15, 16, 22–25, 28–30], and low-density lipoprotein cholesterol (LDL-C) [15, 16, 22–30, 32]. Exenatide, saxagliptin, sitagliptin, liraglutide, rosiglitazone, pioglitazone, beinaglutide, and canagliflozin have been evaluated as add-on drugs to metformin. The details of each drug on lipid parameters are shown in Table 2.

### 3.6. Effects of Antidiabetic Drugs as an Add-On Medication to Metformin on Hormonal Profile

All included researches have investigated the changes in various hormones, including luteinizing hormone (LH) [16, 19, 24–26, 28–32], follicle-stimulating hormone (FSH) [16, 19, 21, 24–26, 28–32], prolactin [19, 21, 31], testosterone [15, 16, 19, 20, 22–32], sex hormone-binding globulin (SHBG) [20, 22–25, 28, 29, 31, 32], dehydroepiandrosterone sulfate (DHEA-S) [16, 20, 22, 23, 25, 27, 29], and progesterone [31]. The impact of all aforementioned drugs has been assessed in different included studies. Table 2 summarizes the details of changes in hormonal profile.

### 3.7. Effects of Antidiabetic Drugs as an Add-On Medication to Metformin on Blood Pressure

Three studies have examined the changes in systolic and diastolic blood pressure (SBP and DBP) [20, 23, 25]. The impact of rosiglitazone [20], saxagliptin [23], and liraglutide [25] has been evaluated as an add-on drug to metformin. Table 2 demonstrates the details of blood pressure alteration following the mentioned drugs.

## 4. Discussion

The current review study aims to find the best choice for an add-on medication to metformin in PCOS patients. Since PCOS is a metabolic disorder associated with an increased risk of multiple metabolic complications, choosing the best combination of antidiabetic drugs in the clinical course of PCOS patients will be very important. Therefore, selecting a second agent as a metformin add-on therapy should be based on the patient's clinical characteristics.

According to the available literature, the impact of different groups of antidiabetic drugs, including DPP4i, SGLT2i, GLP1RA, and TZDs, on glycemic and lipid profiles, anthropometric measures, sexual hormones, and blood pressure was evaluated. As we were unable to conduct a meta-analysis, we determined the best option for combining with metformin based on the consensus of the studies included. The glycemic profile was reported to be affected most by exenatide [22, 27] and saxagliptin [23, 28] as an add-on medication to metformin in PCOS patients. Rosiglitazone influenced the lipid profile of PCOS patients more than other antidiabetic agents [15, 26]. However, HDL-C is reported to increase more among groups consuming metformin alone [15, 28]. It is worth mentioning that rosiglitazone was withdrawn from the European market in 2010 due to an increased risk of heart attacks. The United States Food and Drug Administration (FDA) restricted access to rosiglitazone in 2011. However, the FDA removed its prescribing restrictions in 2013 based on studies that reduced the suspicion of the cardiovascular risks of rosiglitazone [33].

**Figure 2:**
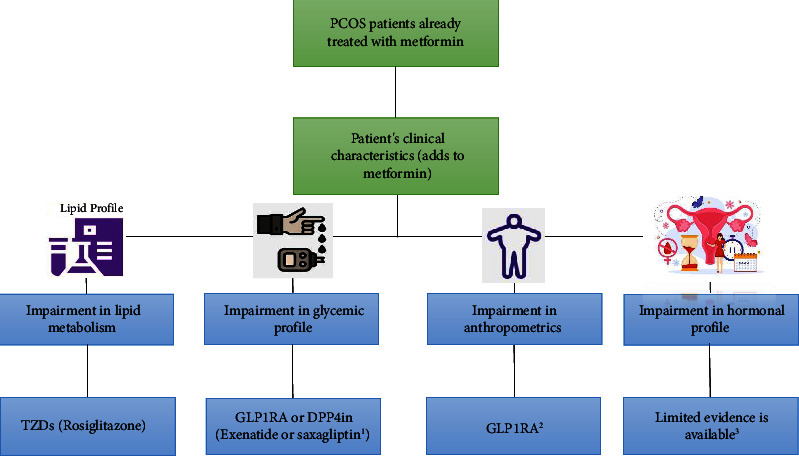
Flow diagram for preferred add-on medications according to the included studies in the systematic review. ^1^Saxagliptin is not yet recommended. ^2^GLP1RA's such as exenatide, liraglutide, and beinaglutide. ^3^However, testosterone levels were more affected by rosiglitazone, liraglutide, beinaglutide, and canagliflozin when used as an additional medication to metformin.

GLP1RA, including exenatide [22, 27], liraglutide [25], and beinaglutide [30], demonstrated to be a good choice in modulating different anthropometric measures such as BW, BMI, WC, and WHR. Results surrounding the efficacy of different antidiabetic drugs in hormonal profile modification are ambiguous. Testosterone is influenced more than other hormones by different antidiabetic drugs such as rosiglitazone [15, 26], liraglutide [31], beinaglutide [30], and canagliflozin [32] as an add-on medication to metformin in PCOS. None of the combined therapies demonstrated a significant change in blood pressure [20, 23, 25].  Figure 2 demonstrates a flowchart for add-on therapies to metformin based on the available literature.

TZDs or glitazones are a group of drugs with insulin-sensitizing properties. TZDs reduce IR in the liver and peripheral tissues by activating the nuclear hormone receptor peroxisome proliferator-activated receptor gamma (PPAR*γ*). They were also reported to affect dyslipidemia state [19, 22] positively. Among patients with T2DM, TZDs, in combination with metformin, were found to be more effective in controlling hyperglycemia than metformin alone. However, metformin monotherapy is more effective in lowering weight [34]. Among PCOS patients, the efficacy of pioglitazone and rosiglitazone, in combination with metformin, has been investigated. Two studies that evaluated pioglitazone showed inconsistent results. Ali et al. found that combination therapy appears to be more effective than metformin monotherapy in improving IR through diminishing interleukin 6 (IL-6) and interleukin 8 (IL-8) levels [19]. While Sohrevardi et al. showed no significant difference between combination therapy and each drug monotherapy [16], results of a Chinese network meta-analysis suggested that a combination therapy is more effective than metformin alone in reducing IR, total testosterone, and TG levels [35]. Rosiglitazone, in addition to metformin, was shown to modulate the lipid profile among obese PCOS patients and is a good choice in patients with severe IR, which do not respond to metformin. Moreover, the combination therapy effectively managed endocrinal abnormalities and modified menstrual patterns among obese patients [15, 26]. However, a study in nonobese PCOS patients without IR found no more beneficial effects than metformin alone [20]. No serious side effects have been reported for pioglitazone or rosiglitazone.

DPP4i are a class of glucose-lowering drugs that act by inhibiting GLP1 degradation. They reduce the serum levels of the DPP4 enzyme by 70–90%, increasing the circulating levels of GLP1 [36]. In patients with T2DM, the combination of DPP4i and metformin reported better glycemic outcomes than metformin alone. However, there is no significant difference in weight change. Beyond these, other add-on medications to metformin, such as TZDs, SGLT2i, and GLP1RA, were more effective in modulating glycemic profile and body weight [34]. Among PCOS patients, two studies evaluated sitagliptin's effectiveness, and two others assessed saxagliptin as an adjunct to metformin. Sitagliptin, in addition to metformin, is reported to be a good choice for improving the fertilization rate but not the pregnancy rate. It exerts its effect by increasing the growth differentiation factor 9 (GDF9) and bone morphogenetic protein 15 (BMP15) expressions [21]. Sitagliptin, in addition to metformin, is more effective than metformin monotherapy in preventing weight regain in PCOS patients who previously consumed liraglutide [24]. The most frequent adverse effect of the individuals consuming sitagliptin and metformin was mild to moderate gastrointestinal complaints. Saxagliptin, in addition to metformin, indicated desirable results in modulating different aspects of prediabetic and diabetic PCOS patients, especially glycemic profile [23, 28]. This combination treatment did not show any additional adverse effects to the gastrointestinal side effects of metformin alone.

GLP1RAs are a group of antidiabetic medications that exert their effect by mimicking the action of the GLP1 hormone. GLP1 and glucose-dependent insulinotropic polypeptide (GIP) are both incretin hormones that stimulate insulin secretion after glucose administration. This medication benefits T2DM patients through different mechanisms, such as increasing insulin excretion, delaying gastric emptying, inhibiting glucagon production, and decreasing pancreatic beta cell apoptosis [37]. According to the half-time, GLP1RAs are categorized into short-acting drugs (half-life of 2–4 hours), such as exenatide and beinaglutide, and long-acting ones (half-time more than 12 hours) such as liraglutide [38]. When added to metformin monotherapy, GLP1RAs, especially long-acting ones, were reported to induce better hypoglycemic effects than other antidiabetic agents, such as DPP4i and SGLT2i [34, 39]. The combination therapy of exenatide and metformin reported better results than metformin alone in modulating anthropometric indexes, insulin sensitivity, and menstrual cycle frequency among overweight and obese PCOS patients [22, 27].

Recently, once weekly subcutaneous injection of semaglutide as a potent GLP1RA approved for long-term weight management has been shown to produce significant weight loss in patients with overweight or obesity and have favorable effects on cardiometabolic risk factors. Also, the FDA's approval of oral semaglutide, the first oral GLP1RA, signals a paradigm shift in treating patients with T2DM. However, to our knowledge, there is no study on semaglutide benefits on the anthropometric factors in combination with metformin in PCOS patients [40].

In addition, the combination therapy indicated higher remission rates of prediabetic PCOS patients than metformin monotherapy (64% and 32%, respectively) [29]. As in all the abovementioned studies, the exenatide was consumed through subcutaneous injections. Pain and itching at the injection site was a common side effect. Mild gastrointestinal reactions were also another common adverse event. Beinaglutide as an adjunct to metformin exerts better short-term effects than metformin alone in modifying different anthropometrics, metabolic, and hormonal profiles [30]. Similar to exenatide, because of subcutaneous administration of beinaglutide, induration and pruritus at the injection site were the common tolerable adverse events. In combination with metformin, liraglutide appears superior to metformin monotherapy in weight loss among obese PCOS cases [25]. This combination was more effective than metformin alone in improving hyperandrogenemia and reproductive disorders. However, combination therapy has no more beneficial effect on modulating glucose metabolism and IR [31]. Mild and moderate gastrointestinal complaints were the most common adverse reactions to this combination therapy.

SGLT2i are medications that primarily block glucose reabsorption in the proximal convoluted tubules, leading to lower blood sugar levels [41]. According to the results of a meta-analysis surrounding adding medications to metformin, SGLT2i was found to be more efficacious than other antidiabetic medications in managing T2DM. Although genital tract infections were more frequent among SGLT2i [42], unfortunately, up to date, only one clinical trial assessed the effectiveness of SGLT2i combined with metformin versus metformin alone. Canagliflozin and metformin exert no different outcomes from metformin monotherapy in weight control, insulin sensitivity, androgen excess, and menstrual frequency [32]. Further investigations are needed to better clarify the efficacy of SGLT2i in addition to metformin among PCOS patients.

It should be noted that the use of any of the mentioned antidiabetic drugs, including TZDs [43], DPP4i [44], GLP1RA [45], and SGLT2i [46], is prohibited during pregnancy, and metformin alone should be prescribed.

Finally, it is important to note that lifestyle modification is one of the pivotal interventions in the management of PCOS patients at early stages [47, 48]. Some studies have demonstrated the greater impact of lifestyle modification than metformin therapy in modulating obesity and menstrual frequency among PCOS patients [49, 50]. Most of the included studies in this systematic review assessed the people with normal diet and physical activity levels and did not measure the impact of lifestyle modification. It is suggested that further investigations assess the effect of lifestyle modification in addition to the abovementioned therapies.

## 5. Strengths, Limitations, and Suggestions

Several review articles are regarding the efficacy of various antidiabetic agents in PCOS patients. However, to the best of our knowledge, this is the first systematic review surrounding the efficacy of an additional medication to metformin. However, there is limited evidence to conduct a meta-analysis, but we have found the best choices as an adjunct for each aspect of PCOS. Further studies on all the abovementioned categories of drugs, especially SGLT2i, are needed to better clarify the best add-on medication to metformin. Besides, all included studies have a 6-month or lower duration of treatment, and we cannot compare the efficacy of the long-term combination therapy and metformin monotherapy. Thus, further long-term trials are needed to discover more accurate results regarding the efficacy and side effects of combination therapies.

## 6. Conclusion

Since PCOS is a metabolic disorder, choosing the best combination of antidiabetic drugs in the clinical course of PCOS patients will be very important. Today, it seems that we need a new metabolic approach for better treatment of these patients.

## Figures and Tables

**Table 1:** Characteristics of the included studies.

Author	Year	Country	Study design	Participants	PCOS diagnostic criteria	Sample size		Age (mean ± SD)		Control	Intervention	Duration (week)	Outcome^*∗*^
						Con	Int	Con	Int				
Ali et al. [19]	2019	Pakistan	Two arms, open-label RCT	PCOS women	ESHRE/ASRM guidelines	53	53	25.6 ± 4.5	25.4 ± 3.7	Met, 1000 mg/day	Met, 1000 mg/day and PGZ 30 mg/day	12	1, 2, 5, 6, 11, 12, 13, 14
Baillargeon et al. [20]	2004	Venezuela	Four arms, double-blind RCT	Nonobese PCOS women with normal insulin sensitivity	NR	28	20	27.7 ± 0.9	27.5 ± 1.1	Met, 850 mg/day	Met, 850 mg/day and RSG 4 mg/day	24	1, 2, 4, 5, 14, 15, 16, 18, 19
Daneshjou et al. [21]	2022	Iran	Four arms, double-blind RCT	PCOS women candidate for ICSI	ESHRE/ASRM guidelines	15	15	28.9 ± 2.7	30.2 ± 3.1	Met, 1000 mg/day	Met, 1000 mg/day and SITA 100 mg/day	8	6, 12, 13, 16
Elkind-Hirsch et al. [22]	2008	USA	Three arms, open-label RCT	Overweight and obese PCOS women	Rotterdam criteria	20	20	27.7 ± 1.3	32.1 ± 0.7	Met, 2000 mg/day	Met, 2000 mg/day and EX 20 *μ*g/day	24	1, 2, 6, 7, 8, 9, 10, 14, 15, 16
Elkind-Hirsch et al. [23]	2017	USA	Three arms, single-blind RCT	Prediabetic PCOS women	NIH 1990 criteria	12	11	29.9 ± 7	29.6 ± 8	Met, 2000 mg/day	Met, 2000 mg/day and SAXA 5 mg/day	16	2, 3, 5, 6, 7, 8, 9, 10, 14, 15, 16, 18, 19
Ferjan et al. [24]	2017	Slovenia	Two arms, open-label RCT	Obese PCOS women pretreated with LIR	NR	12	12	34.3 ± 6.8		MET, 2000 mg/day	Met, 2000 mg/day and SITA 100 mg/day	12	1, 2, 3, 6, 7, 8, 9, 10, 11, 12, 14, 15
Sever et al. [25]	2014	Slovenia	Three arms, open-label RCT	Obese PCOS women pretreated with met	NR	14	11	31.3 ± 9.4	31.1 ± 5.5	Met, 2000 mg/day	Met, 2000 mg/day and LIR 1.2 mg/day	12	1, 2, 3, 6, 7, 8, 9, 10, 11, 12, 14, 15, 16, 18, 19
Li et al. [15]	2020	China	Three arms, open-label RCT	Obese PCOS women	Rotterdam criteria	68	69	25.8 ± 4.4	25.9 ± 4	Met, 1500 mg/day	Met, 1000 mg/day and RSG 4 mg/day	24	1, 2, 3, 4, 5, 6, 7, 8, 9, 10, 14
Liao et al. [26]	2011	China	Three arms, open-label RCT	Obese PCOS women	Rotterdam criteria	28	27	27.7 ± 2.9	27 ± 3.2	Met, 1500 mg/day	Met, 1500 mg/day and RSG 4 mg/day	24	5, 6, 7, 9, 11, 12, 14
Ma et al. [27]	2021	China	Two arms, open-label RCT	Overweight and obese PCOS	Rotterdam criteria	25	25	28.1 ± 4.4	30.1 ± 4.5	Met, 1500 mg/day	Met, 1500 mg/day and EX 2 mg/week	12	1, 2, 3, 5, 6, 7, 8, 9, 10, 14, 16
Sohrevardi et al. [16]	2016	Iran	Three arms, open-label RCT	PCOS women	Rotterdam criteria	22	23	28.7 ± 6.3	30.7 ± 6.1	Met, 1500 mg/day	Met, 1500 mg/day and PGZ 30 mg/day	12	1, 2, 4, 5, 6, 7, 8, 9, 10, 11, 12, 14, 16
Tao et al. [28]	2018	China	Three arms, open-label RCT	PCOS women with new-onset T2DM	Rotterdam criteria	21	21	28 ± 3	29 ± 5	Met, 2000 mg/day	Met, 2000 mg/day and SAXA 5 mg/day	24	1, 2, 3, 4, 5, 6, 7, 8, 9, 10, 11, 12, 14, 15, 20
Tao et al. [29]	2021	China	Three arms, open-label RCT	Overweight and obese prediabetic PCOS women	Rotterdam criteria	50	50	NR	NR	Met, 2000 mg/day	Met, 2000 mg/day and EX 20 *μ*g/day	12	1, 2, 7, 8, 9, 10, 11, 12, 14, 15, 16
Wen et al. [30]	2023	China	Two arms, open-label RCT	Obese PCOS women	Rotterdam criteria	30	30	25.4 ± 3.1	26.7 ± 4.4	Met, 1700 mg/day	Met, 1700 mg/day and beinaglutide 0.6 mg/day	12	1, 2, 3, 4, 5, 6, 7, 8, 9, 10, 11, 12, 14
Xing et al. [31]	2022	China	Two arms, open-label RCT	Overweight PCOS women	Rotterdam criteria	25	27	23.5 ± 4.6	25.8 ± 4.4	Met, 2000 mg/day	Met, 2000 mg/day and LIR 1.2 mg/day	4 & 12	1, 2, 5, 6, 11, 12, 13, 14, 15, 17
Zhang et al. [32]	2022	China	Two arms, open-label RCT	Overweight and obese PCOS women	Rotterdam criteria	20	21	25.5 ± 4.3	26.3 ± 5.8	Met, 2000 mg/day	Met, 2000 mg/day and CANA 100 mg/day	12	1, 2, 5, 6, 7, 8, 9, 11, 12, 14, 15

^*∗*^Outcomes are summarized as follows: 1, weight; 2, body mass index; 3, waist circumference; 4, waist-to-hip ratio; 5, fasting blood sugar; 6, HOMA-IR; 7, triglyceride; 8, total cholesterol; 9, LDL-C; 10, HDL-C; 11, LH; 12, FSH; 13, prolactin; 14, testosterone; 15, SHBG; 16, DHEA-S; 17, progesterone; 18, systolic blood pressure; 19, diastolic blood pressure; 20, HbA1C. RCT, randomized clinical trial; PCOS, polycystic ovary syndrome; Con, control; Int, intervention; NR, not reported; ICSI, intracytoplasmic sperm injection; Met, metformin; PGZ, pioglitazone; RSG, rosiglitazone; SITA, sitagliptin; EX, exenatide; SAXA, saxagliptin; LIR, liraglutide; CANA, canagliflozin.

**Table 2:** Changes in different aspects of PCOS patients following different antidiabetic drugs as an add-on medication to metformin.

		Pioglitazone [19]				Pioglitazone [16]				Rosiglitazone [20]				Rosiglitazone [15]				Rosiglitazone [26]				Canagliflozin [32]			
		Control		Intervention		Control		Intervention		Control		Intervention		Control		Intervention		Control		Intervention		Control		Intervention	
		Met, 1000 mg/day		Met, 1000 mg/day + PGZ 30 mg/day		Met, 1500 mg/day		Met, 1500 mg/day + PGZ 30 mg/day		Met, 850 mg/day		Met, 850 mg/day + RSG 4 mg/day		Met, 1500 mg/day		Met, 1000 mg/day + RSG 4 mg/day		Met, 1500 mg/day		Met, 1500 mg/day + RSG 4 mg/day		Met, 2000 mg/day		Met, 2000 mg/day + CANA 100 mg/day	
		Pre	Post^1^	Pre	Post^1^	Pre	Post^1^	Pre	Post^1^	Pre	Post^2^	Pre	Post^2^	Pre	Post^2^	Pre	Post^2^	Pre	Post^2^	Pre	Post^2^	Pre	Post^1^	Pre	Post^1^

Glycemic profile	FBS (mg/dL)	90.9 (7)	91.6 (6.6)	92.5 (5.8)	92 (6.1)	97.2 (9)	91.8 (5.4)^*∗*^	91.8 (9)	90 (7.2)	86.8 (2.4)	84.4 (2.4)	79.1 (2.9)	84.7 (2.8)	96.8 (7.4)	91.6 (6.1)^*∗*^	98.4 (7.9)	92.5 (10.4)^*∗*^	75.6 (18)	73.8 (18)	81 (14.4)	70.2 (12.6)	95.4	95.4	102.6	93.6^*∗*^
	HOMA-IR	7.1 (3)	3.9 (2)^*∗*^	6.2 (2.6)	3.8 (2.1)^*∗*^	4.2 (2.7)	2.3 (1.2)^*∗*^	2.7 (1.8)	1.6 (0.8)^*∗*^	NR	NR	NR	NR	5.4 (1.9)	3.6 (1.3)^*∗*^	5.5 (2)	3.6 (1.5)^*∗*^	5 (1.9)	2.9 (1.3)^*∗*^	5.4 (2)	2.2 (1.2)^*∗*^	4.25	3.51^*∗*^	5.7	3.14^*∗*^

Anthropometric measures	BW (kg)	76.7 (16.8)	76.5 (16.3)^*∗*^	75.7 (12.9)	74.6 (12)^*∗*^	71.3 (11.2)	71 (12.8)	72.6 (9.4)	71.5 (9.8)	62.1 (0.6)	61.4 (0.3)	62.1 (0.7)	62.3 (0.4)	70 (8)	63.2 (7)^*∗*^	70 (8.2)	66.4 (8)^*∗*^	NR	NR	NR	NR	74.7 (8.9)	72.5 (10)^*∗*^	81.2 (9.8)	75.4 (8.7)^*∗*^
	BMI	30.1 (6.6)	29.9 (6.5)^*∗*^	28.8 (5.1)	28.3 (4.6)^*∗*^	27.5 (3.6)	27.4 (4.4)	28.5 (3.2)	28 (3.4)	24.6 (0.2)	24.3 (0.1)	24.6 (0.3)	24.6 (0.1)	27.7 (2)	25 (1.8)^*∗*^	27.3 (2.1)	25.9 (2.2)^*∗*^	NR	NR	NR	NR	29.3 (3.2)	27.1 (3.5)^*∗*^	31.1 (3)	28.6 (2.9)^*∗*^
	WC (cm)	NR	NR	NR	NR	NR	NR	NR	NR	NR	NR	NR	NR	93.4 (7.2)	89.5 (7.5)^*∗*^	92.5 (7.5)	90.2 (7)^*∗*^	NR	NR	NR	NR	NR	NR	NR	NR
	WHR	NR	NR	NR	NR	0.83 (0.03)	0.83 (0.04)	0.82 (0.04)	0.82 (0.05)	0.8 (0.01)	0.8	0.81 (0.01)	0.8	0.93 (0.06)	0.89 (0.06)^*∗*^	0.93 (0.06)	0.9 (0.06)^*∗*^	NR	NR	NR	NR	NR	NR	NR	NR

Lipid profile	TG (mg/dL)	NR	NR	NR	NR	140.3 (58.2)	147 (78.9)	131 (59.3)	114.3 (78.5)	NR	NR	NR	NR	160.1 (53.1)	130 (53)^*∗*^	162 (54)	114.1 (60)^*∗*^	327.4 (70.8)	194.7 (62)^*∗*^	327.4 (44.2)	177 (53.1)^*∗*^	131.8	126.5	136.3	106.1^*∗*^
	TC (mg/dL)	NR	NR	NR	NR	215 (36.5)	209.7 (37.2)	196 (36.6)	197 (35.6)	NR	NR	NR	NR	175.3 (35.5)	158.3 (42.4)^*∗*^	173.7 (33.6)	145.9 (29.3)^*∗*^	NR	NR	NR	NR	183 (24.3)	175.3 (20)	189.2 (36)	175.3 (30.9)^*∗*^
	HDL-C (mg/dL)	NR	NR	NR	NR	51.1 (12.1)	52.9 (11)	52.2 (12.2)	55.7 (13.2)	NR	NR	NR	NR	49.8 (7.34)	57.1 (9.27)^*∗*^	50.5 (10)	53.3 (10.4)^*∗*^	NR	NR	NR	NR	NR	NR	NR	NR
	LDL-C (mg/dL)	NR	NR	NR	NR	131.8 (26.7)	127 (28)	126.9 (33.5)	124 (29.8)	NR	NR	NR	NR	112.7 (30.5)	86.5 (31.6)^*∗*^	113.5 (22.4)	85.7 (30.9)^*∗*^	104.2 (11.6)	88.8 (7.7)^*∗*^	104.2 (11.6)	73.3 (7.7)^*∗*^	116.2 (20.8)	109.2 (18.9)	118.1 (37.4)	109.2 (27)

Hormonal profile	LH (IU/L)	5.7 (3.7)	4.9 (2.2)	6.6 (4.49)	5.1 (2.5)^*∗*^	7.9 (2.3)	6.3 (2.4)^*∗*^	6.8 (2.7)	6.4 (2.2)	NR	NR	NR	NR	NR	NR	NR	NR	11.4 (2.1)	4 (1.2)^*∗*^	12.1 (2.2)	3.2 (0.2)^*∗*^	11.6	10.27	10.8	8.59
	FSH (IU/L)	8.4	6.2 (3.8)^*∗*^	8.7 (6.7)	6.1 (3.8)^*∗*^	5.2 (1.7)	5.5 (1.4)	5.6 (2.2)	7 (2.2)^*∗*^	NR	NR	NR	NR	NR	NR	NR	NR	3.5 (1.5)	3.3 (1.6)	3.6 (1.4)	3.3 (1.4)	6 (1.6)	5.36 (1.9)	6.6 (1.5)	5.84 (2.2)
	Prolactin (ng/mL)	376.5 (185.4)	261.2 (131.1)^*∗*^	239.4 (136.6)	199.4 (96.4)^*∗*^	NR	NR	NR	NR	NR	NR	NR	NR	NR	NR	NR	NR	NR	NR	NR	NR	NR	NR	NR	NR
	Testosterone (ng/mL)	0.32 (0.1)	0.19 (0.09)^*∗*^	0.28 (0.14)	0.18 (0.07)^*∗*^	0.7 (0.2)	0.6 (0.18)^*∗*^	0.6 (0.2)	0.6 (0.1)	1.1 (0.11)	0.37 (0.04)	1.9 (0.13)	0.41 (0.06)	0.66 (0.17)	0.5 (0.18)^*∗*^	0.65 (0.25)	0.46 (0.19)^*∗*^	0.57 (0.08)	0.46 (0.08)^*∗*^	0.57 (0.11)	0.37 (0.11)^*∗*^	0.89	0.71^*∗*^	0.95	0.53^*∗*^
	DHEA-S (mg/L)	NR	NR	NR	NR	1.5 (0.7)	1.4 (0.5)	1.4 (0.7)	1.5 (0.8)	3.3 (0.35)	3 (0.39)	2.9 (0.41)	4.1 (0.51)	NR	NR	NR	NR	NR	NR	NR	NR	NR	NR	NR	NR

BP	SBP (mmHg)	NR	NR	NR	NR	NR	NR	NR	NR	123.9 (0.6)	119.5 (0.7)	123.2 (0.7)	118.7 (0.8)	NR	NR	NR	NR	NR	NR	NR	NR	NR	NR	NR	NR
	DBP (mmHg)	NR	NR	NR	NR	NR	NR	NR	NR	82.9 (0.6)	81.4 (0.5)	83.6 (0.7)	81.5 (0.6)	NR	NR	NR	NR	NR	NR	NR	NR	NR	NR	NR	NR

		*Sitagliptin* [21]				*Sitagliptin* [24]				*Exenatide* [22]				*Exenatide* [27]				*Exenatide* [29]							
		Control		Intervention		Control		Intervention		Control		Intervention		Control		Intervention		Control		Intervention					
		Met 1000 mg/day		Met, 1000 mg/day + SITA 100 mg/day		Met, 2000 mg/day		Met, 2000 mg/day + SITA 100 mg/day		Met, 2000 mg/day		Met, 2000 mg/day + EX 20 *μ*g/day		Met, 1500 mg/day		Met, 1500 mg/day + EX 2 mg/week		Met, 2000 mg/day		Met, 2000 mg/day + EX 20 *μ*g/day					
		Pre	Post^3^	Pre	Post^3^	Pre	Post^1^	Pre	Post^1^	Pre	Post^2^	Pre	Post^2^	Pre	Post^1^	Pre	Post^1^	Pre	Post^1^	Pre	Post^1^				

Glycemic profile	FBS (mg/dL)	NR	NR	NR	NR	NR	NR	NR	NR	NR	NR	NR	NR	93.6 (8.3)	93.4 (9.2)	93.8 (10.2)	88.7 (8.4)^*∗*^	NR	NR	NR	NR				
	HOMA-IR	4.09 (0.71)	3.43 (0.47)^*∗*^	3.86 (0.79)	3.39 (0.61)^*∗*^	6.1 (7.1)	2.9 (1.8)	3.1 (1.3)	2.1 (1.5)	6.03 (0.96)	5.7 (0.7)	4.3 (1)	3.5 (0.7)	4.49 (1.1)	4.8 (2.1)	5.3 (3)	4.7 (1.5)	NR	NR	NR	NR				

Anthropometric measures	BW (kg)	NR	NR	NR	NR	101.2 (12.1)	105.9 (13.7)^*∗*^	100.4 (12.9)	101.4 (14)	113.4 (7)	111.8 (6)	112 (8)	106.4 (6)	79.1 (10.8)	77.05 (9.75)^*∗*^	82.34 (11.42)	78.57 (10.94)^*∗*^	80 (8.8)	76 (10)^*∗*^	83.2 (13)	76.6 (13.2)^*∗*^				
	BMI	NR	NR	NR	NR	37.8 (4.7)	39.5 (5)^*∗*^	34.8 (5.4)	35.1 (5.7)	43.3 (2)	42.3 (2)	40.9 (2)	39.2 (2)	30.4 (3.16)	29.63 (2.8)^*∗*^	30.8 (3.41)	29.4 (3.32)^*∗*^	29.64 (3.6)	28.2 (3.6)^*∗*^	31.6 (4.67)	29.17 (4.8)^*∗*^				
	WC (cm)	NR	NR	NR	NR	106.8 (9.5)	108.8 (12.8)	105.3 (12.7)	103.3 (12.5)	NR	NR	NR	NR	96.6 (9.1)	95 (8.1)^*∗*^	97.3 (9.6)	92.7 (8.7)^*∗*^	NR	NR	NR	NR				
	WHR	NR	NR	NR	NR	NR	NR	NR	NR	NR	NR	NR	NR	NR	NR	NR	NR	NR	NR	NR	NR				

Lipid profile	TG (mg/dL)	NR	NR	NR	NR	115 (44.2)	123.9 (44.2)	132.7 (61.9)	141.6 (70.8)	155 (15)	188 (17)	135 (15)	126 (17)	150.4	217.7^*∗*^	115	177^*∗*^	137.1 (97.3)	122.1 (11.5)^*∗*^	176.1 (0.67)	105.3 (49.5)^*∗*^				
	TC (mg/dL)	NR	NR	NR	NR	185.3 (38.6)	189.2 (42.4)	189.2 (38.6)	189.2 (42.4)	188 (9)	197 (9)	212 (9)	196 (9)	198.8 (33.6)	217.7 (34)^*∗*^	190.7 (30.1)	198 (38.2)	177.2 (32)	179.9 (7.7)	181.8 (29.3)	177.6 (25.4)				
	HDL-C (mg/dL)	NR	NR	NR	NR	50.2 (7.7)	54 (7.7)	46.33 (7.7)	50.2 (7.7)^*∗*^	41.4 (2)	39.7 (1.7)	46.8 (2)	46.1 (1.7)	44 (8.5)	56 (13.9)^*∗*^	45.9 (9.2)	55.6 (13.1)^*∗*^	45.1 (5.8)	47.8 (2.8)	43.6 (6.3)	43.2 (7.14)				
	LDL-C (mg/dL)	NR	NR	NR	NR	112 (34.7)	112 (34.7)	115.8 (34.7)	119.7 (30.9)	115.6 (8)	119.5 (8)	139.2 (8)	124.6 (8)	130.5 (27.4)	130.1 (30.1)	124.3 (23.5)	115 (32)	116.6 (25.5)	97.3 (14.6)^*∗*^	129.7 (28.2)	108.5 (19.7)^*∗*^				

Hormonal profile	LH (IU/L)	NR	NR	NR	NR	6.4 (4.3)	5.8 (2.7)	7.9 (8.1)	8.8 (14.5)	NR	NR	NR	NR	NR	NR	NR	NR	7.8 (5.6)	6.1 (2.6)^*∗*^	7.5 (3.7)	6.5 (2.2)^*∗*^				
	FSH (IU/L)	4.99 (0.72)	5.98 (0.96)	5.31 (1.1)	5.83 (0.88)	3.6 (1.6)	4.9 (2)^*∗*^	5.5 (2.3)	5.2 (3)	NR	NR	NR	NR	NR	NR	NR	NR	6.3 (1.5)	6.2 (1.8)	6.4 (1.9)	6.5 (1)				
	Prolactin (ng/mL)	13.56 (0.45)	14.7 (0.69)	14.25 (0.69)	15.63 (1.15)	NR	NR	NR	NR	NR	NR	NR	NR	NR	NR	NR	NR	NR	NR	NR	NR				
	Testosterone (ng/mL)	NR	NR	NR	NR	0.51 (0.17)	0.4 (0.17)	0.37 (0.17)	0.37 (0.2)	0.56 (0.08)	0.53 (0.07)	0.59 (0.08)	0.41 (0.07)	0.78 (0.22)	0.56 (0.2)^*∗*^	0.74 (0.29)	0.57 (0.25)^*∗*^	0.63 (0.28)	0.53 (0.23)^*∗*^	0.67 (0.21)	0.54 (0.27)^*∗*^				
	DHEA-S (mg/L)	2.29 (0.72)	0.66 (0.09)	2.81 (1.18)	0.58 (0.12)	NR	NR	NR	NR	1.42 (0.19)	1.61 (0.2)	1.23 (0.2)	1.21 (0.2)	2.65 (1.2)	2.61 (1.2)	2.63 (1.2)	2.61 (1.3)	2.55 (1.1)	2.46 (0.53)	2.48 (0.81)	2.34 (0.66)				

BP	SBP (mmHg)	NR	NR	NR	NR	NR	NR	NR	NR	NR	NR	NR	NR	NR	NR	NR	NR	NR	NR	NR	NR				
	DBP (mmHg)	NR	NR	NR	NR	NR	NR	NR	NR	NR	NR	NR	NR	NR	NR	NR	NR	NR	NR	NR	NR				

		*Saxagliptin* [23]				*Saxagliptin* [28]				*Liraglutide* [25]				*Liraglutide* [31]						*Beinaglutide* [30]					
		Control		Intervention		Control		Intervention		Control		Intervention		Control			Intervention			Control		Intervention			
		Met, 2000 mg/day		Met, 2000 mg/day + SAXA 5 mg/day		Met, 2000 mg/day		Met, 2000 mg/day + SAXA 5 mg/day		Met, 2000 mg/day		Met, 2000 mg/day + LIR 1.2 mg/day		Met, 2000 mg/day			Met, 2000 mg/day + LIR 1.2 mg/day			Met, 1700 mg/day		Met, 1700 mg/day + beinaglutide 0.6 mg/day			
		Pre	Post^4^	Pre	Post^4^	Pre	Post^2^	Pre	Post^2^	Pre	Post^1^	Pre	Post^1^	Pre	Post^5^	Post^1^	Pre	Post^5^	Post^1^	Pre	Post^1^	Pre	Post^1^		

Glycemic profile	FBS (mg/dL)	100.8 (10.2)	97.2 (12.6)	100.8 (9.9)	90 (6.5)	101.1	90.1^*∗*^	105.1	92.5^*∗*^	NR	NR	NR	NR	98.6 (9.3)	NR	93.6 (5.7)^*∗*^	102.6 (16.7)	NR	90.9 (7.2)^*∗*^	95.2 (5)	93.8 (15.1)^*∗*^	99.1 (5.9)	92.1 (5.2)^*∗*^		
	HOMA-IR	6.8 (3.6)	5.9 (3.7)	5 (2)	3.6 (2.1)	3.56	2.29^*∗*^	4.22	2.45^*∗*^	3.8 (2.8)	2.5 (1.7)	1.7 (3.6)	2.1 (2.1)	4.53 (3.07)	NR	2.86 (2.05)^*∗*^	4.85 (2.09)	NR	2.62 (1.05)^*∗*^	5.39 (1.86)	4.96 (2.74)	5.68 (1.94)	4.98 (1.73)^*∗*^		

Anthropometric measures	BW (kg)	NR	NR	NR	NR	67.9	65.1^*∗*^	69.3	67^*∗*^	103.2 (6.3)	102 (6.8)	105.5 (20.6)	99 (21.2)	76.5 (12.4)	74.1 (12.4)^*∗*^	71.4 (11.5)^*∗*^	79 (8.4)	73.2 (7.7)^*∗*^	69.9 (7.2)^*∗*^	72.5 (5.9)	70 (3.8)^*∗*^	72.9 (6.8)	68.4 (5.9)^*∗*^		
	BMI	42.1 (7.3)	42 (7.7)	43.8 (10.5)	42 (10.2)	26.4	25.32^*∗*^	26.38	25.46^*∗*^	36.6 (3.5)	36.1 (3.8)	37.6 (5.1)	35.3 (5.5)	28.8 (4.2)	27.9 (4.2)^*∗*^	26.8 (3.7)^*∗*^	29.6 (3.4)	27.4 (3)^*∗*^	26.2 (2.7)^*∗*^	29 (2.3)	27 (1)^*∗*^	28.8 (2.9)	25.9 (2.7)^*∗*^		
	WC (cm)	111 (11)	109 (13)	111 (15)	106 (16)	82.8	79.9^*∗*^	84.7	81.5^*∗*^	122.3 (7)	120.7 (7.8)	121.9 (17.7)	116.4 (18.4)	NR	NR	NR	NR	NR	NR	89.7 (5.4)	89.9 (5.7)	96.8 (6.9)	94.6 (5.4)^*∗*^		
	WHR	NR	NR	NR	NR	0.85 (0.06)	0.83 (0.05)^*∗*^	0.86 (0.06)	0.83 (0.05)^*∗*^	NR	NR	NR	NR	NR	NR	NR	NR	NR	NR	0.95 (0.13)	0.94 (0.12)	0.99 (0.06)	0.98 (0.06)		

Lipid profile	TG (mg/dL)	134.5 (56.6)	164.6 (61.9)	142.5 (53.1)	117.7 (35.4)	116.8	79.6^*∗*^	118.5	84^*∗*^	141.2 (32.7)	115 (32.7)	123.9 (26.5)	123.9 (32.7)	NR	NR	NR	NR	NR	NR	230 (66.3)	215 (48.6)^*∗*^	255.7 (72.5)	237.1 (65.5)^*∗*^		
	TC (mg/dL)	189.1 (27)	181.4 (23.9)	189.1 (29.3)	177.6 (20.8)	190.7	176^*∗*^	185.3	174.5^*∗*^	181.5 (23.1)	177.6 (15.4)	204.6 (42.4)	177.6 (30.9)	NR	NR	NR	NR	NR	NR	174.5 (24.3)	171.8 (32)	185.7 (17.3)	184.1 (18.5)		
	HDL-C (mg/dL)	43.2 (13.5)	40.9 (11.6)	40.1 (9.6)	40.9 (7.7)	52.1	54.4^*∗*^	47.8	51	42.4 (7.7)	46.3 (7.7)	50.2 (15.4)	42.4 (7.7)	NR	NR	NR	NR	NR	NR	43.6 (16.6)	50.9 (12.3)^*∗*^	41.3 (7.3)	42.8 (9.2)		
	LDL-C (mg/dL)	118.1 (23.1)	108.8 (23.1)	122 (21.6)	113.9 (19.3)	128.1 (26.6)	111.5 (17.3)^*∗*^	131.2 (28.1)	115 (15.8)^*∗*^	112 (23.1)	112 (19.3)	131.3 (30.9)	112 (30.9)	NR	NR	NR	NR	NR	NR	114.3 (13.9)	112.3 (11.2)	117.3 (14.6)	115 (20.4)		

Hormonal profile	LH (IU/L)	NR	NR	NR	NR	11.74	7.55^*∗*^	9.62	7.68	NR	NR	NR	NR	11.97 (4.2)	12.08 (6.47)	9.77 (5.81)	12.09 (5.2)	9.54 (4.5)	6.61 (4.7)^*∗*^	10 (3)	10.4 (2.5)	10.8 (4.6)	10.1 (3.9)		
	FSH (IU/L)	NR	NR	NR	NR	8.18	5.92^*∗*^	7.66	5.97^*∗*^	NR	NR	NR	NR	6.03 (1.48)	5.91 (1.72)	5.28 (2.11)	6.22 (1.4)	5.15 (1.9)^*∗*^	4.4 (2.6)^*∗*^	5.8 (1.1)	5.9 (0.8)	5.7 (1)	5.8 (0.9)		
	Prolactin (ng/mL)	NR	NR	NR	NR	NR	NR	NR	NR	NR	NR	NR	NR	10.8 (4.2)	10.8 (4.5)	13 (5.7)^*∗*^	10.2 (3.6)	10.6 (4.6)	12.9 (4.9)^*∗*^	NR	NR	NR	NR		
	Testosterone (ng/mL)	0.42 (0.17)	0.31 (0.12)	0.42 (0.18)	0.31 (0.21)	0.74	0.6^*∗*^	0.75	0.65^*∗*^	0.49 (0.28)	0.43 (0.26)	0.6 (0.37)	0.46 (0.2)	0.84 (0.25)	0.81 (0.35)	0.79 (0.31)	0.79 (0.2)	0.7 (0.2)	0.62 (0.2)^*∗*^	NR	NR	NR	NR		
	DHEA-S (mg/L)	1.76 (0.55)	1.7 (0.55)	1.46 (0.81)	1.4 (0.66)	NR	NR	NR	NR	2.28 (1.17)	2.13 (1.32)	2.1 (0.77)	1.95 (0.88)	NR	NR	NR	NR	NR	NR	NR	NR	NR	NR		

BP	SBP (mmHg)	135.7 (7)	133 (11)	131.6 (12)	131 (13)	NR	NR	NR	NR	121.9 (13.1)	116.8 (14.6)	125 (13)	126.7 (18.2)	NR	NR	NR	NR	NR	NR	NR	NR	NR	NR		
	DBP (mmHg)	88.6 (8)	85.4 (9.8)	82.5 (13)	83.5 (8.9)	NR	NR	NR	NR	74.1 (12.2)	70.2 (9.4)	80 (6.6)	79.9 (7.8)	NR	NR	NR	NR	NR	NR	NR	NR	NR	NR		

^1^After 12 weeks of intervention. ^2^After 24 weeks of intervention. ^3^After 8 weeks of intervention. ^4^After 16 weeks of intervention. ^5^After 4 weeks of intervention. ^*∗*^Significant differences between before and after intervention. Values are mean (standard deviation) if available. Pre, before intervention; post, after intervention; FBS, fasting blood sugar; BW, body weight; BMI, body mass index; WC, waist circumference; WHR, waist-to-hip ratio; TG, triglyceride; TC, total cholesterol; SBP, systolic blood pressure; DBP, diastolic blood pressure; Met, metformin; PGZ, pioglitazone; RSG, rosiglitazone; CANA, canagliflozin; SITA, sitagliptin; EX, exenatide; SAXA, saxagliptin; LIR, liraglutide; NR, not reported.

## Data Availability

The data supporting this study are from previously reported studies and datasets, which have been cited.

## Supplementary Materials

Supplementary Materials Supplementary File 1: details of search line for each database. Supplementary File 2: details of quality assessment process.
