# Supplementary material for: Comparative Effectiveness of Antidiabetic Drugs as an Additional Therapy to Metformin in Women with Polycystic Ovary Syndrome: A Systematic Review of Metabolic Approaches
Source: Int J Endocrinol. 2024 Mar 11;2024:9900213. doi: 10.1155/2024/9900213 (PMC10948218; doi:10.1155/2024/9900213)
Supplement: Supplementary Materials — Supplementary File 1: details of search line for each database. Supplementary File 2: details of quality assessment process. [file 9900213.f1.zip › Supplementary file 1.pdf]

**Supplementary file 1: Details of search line for each database**

**PubMed:**

(“Polycystic Ovary Syndrome”[Mesh] OR “PCOS” OR “Polycystic Ovarian Syndrome” OR “Stein Leventhal Syndrome” OR “Stein-Leventhal Syndrome” OR “Polycystic Ovar*”) AND (“Metformin”[Mesh] OR Metformin)

*With the Clinical Trial filter, 505 records were found.*

**Scopus:**

(“Polycystic Ovary Syndrome” OR “PCOS” OR “Polycystic Ovarian Syndrome” OR “Stein Leventhal Syndrome” OR “Stein-Leventhal Syndrome” OR “Polycystic Ovar*”) AND (Metformin) AND (“clinical trial” OR “randomized clinical trial” OR “RCT” OR “trial”)

*With the title/abstract and keyword filter 1445 records were found.*

**Embase:**

(‘Polycystic Ovary Syndrome’ OR ‘PCOS’ OR ‘Polycystic Ovarian Syndrome’ OR ‘Stein Leventhal Syndrome’ OR ‘Stein-Leventhal Syndrome’ OR ‘Polycystic Ovar*’) AND (Metformin)

*With the Controlled Clinical Trial filter, 797 records were found.*

**Web of Science:**

(“Polycystic Ovary Syndrome” OR “PCOS” OR “Polycystic Ovarian Syndrome” OR “Stein Leventhal Syndrome” OR “Stein-Leventhal Syndrome” OR “Polycystic Ovar*”) AND (Metformin) AND (“clinical trial” OR “randomized clinical trial” OR “RCT” OR “trial”)

*With the topic filter (title/abstract and keyword) 741 records were found.*
