# Supplementary material for: Comparative Effectiveness of Antidiabetic Drugs as an Additional Therapy to Metformin in Women with Polycystic Ovary Syndrome: A Systematic Review of Metabolic Approaches
Source: Int J Endocrinol. 2024 Mar 11;2024:9900213. doi: 10.1155/2024/9900213 (PMC10948218; doi:10.1155/2024/9900213)
Supplement: Supplementary Materials — Supplementary File 1: details of search line for each database. Supplementary File 2: details of quality assessment process. [file 9900213.f1.zip › Supplementary file 2.pdf]

Supplementary file 2: Details of quality assessment process

Study	Questions of NIH quality assessment tool for controlled intervention studies[1]														Summary quality[2]
	1	2	3	4	5	6	7	8	9	10	11	12	13	14	
Ali et al 2019	Yes	Yes	No	No	No	Yes	Yes	Yes	Yes	Yes	Yes	Yes	Yes	Yes	Good
Baillargeon et al 2004	Yes	Yes	Yes	Yes	No	No	Yes	Yes	Yes	Yes	Yes	Yes	NR	Yes	Good
Daneshjou et al 2022	Yes	Yes	Yes	Yes	No	Yes	No	Yes	Yes	Yes	Yes	Yes	Yes	Yes	Good
Elkind-Hirsch et al 2008	Yes	Yes	No	No	No	Yes	No	Yes	Yes	Yes	Yes	Yes	Yes	Yes	Good
Elkind-Hirsch et al 2017	Yes	Yes	No	No	Yes	Yes	Yes	Yes	Yes	Yes	Yes	Yes	NR	Yes	Good
Ferjan et al 2017	Yes	No	No	No	No	Yes	Yes	Yes	Yes	Yes	Yes	NR	Yes	Yes	Fair
Jensterle Sever et al 2014	Yes	Yes	No	No	No	Yes	Yes	Yes	Yes	Yes	Yes	NR	Yes	Yes	Good
Li et al 2020	Yes	Yes	No	No	No	Yes	Yes	Yes	Yes	Yes	Yes	Yes	Yes	Yes	Good
Liao et al 2011	Yes	No	No	No	No	NR	Yes	Yes	Yes	Yes	Yes	Yes	NR	Yes	Fair
Ma et al 2021	Yes	Yes	No	No	No	Yes	No	Yes	Yes	Yes	Yes	Yes	Yes	Yes	Good
Sohrevardi et al 2016	Yes	Yes	No	No	No	NR	No	Yes	Yes	Yes	Yes	NR	Yes	Yes	Fair
Tao et al 2018	Yes	No	No	No	No	Yes	Yes	Yes	Yes	Yes	Yes	Yes	Yes	Yes	Good
Tao et al 2021	Yes	Yes	No	No	No	Yes	Yes	Yes	Yes	Yes	Yes	Yes	NR	Yes	Good
Wen et al 2023	Yes	Yes	No	No	No	Yes	Yes	Yes	Yes	Yes	Yes	Yes	Yes	Yes	Good
Xing et al 2022	Yes	Yes	No	No	No	Yes	No	Yes	Yes	Yes	Yes	Yes	Yes	Yes	Good
Zhang et al 2022	Yes	Yes	No	No	No	Yes	No	Yes	Yes	Yes	Yes	No	Yes	Yes	Fair

1: Available online at: https://www.nhlbi.nih.gov/health-topics/studyquality-assessment-tools
2: For each paper, a total score of 0-4 out of 14 was rated as poor quality, 5-9 out of 14 as fair quality, and 10-14 out of 14 as good quality
